# The Ctf18RFC Clamp Loader Is Essential for Telomere Stability in Telomerase-Negative and *mre11* Mutant Alleles

**DOI:** 10.1371/journal.pone.0088633

**Published:** 2014-02-12

**Authors:** Honghai Gao, Daniel L. Moss, Courtney Parke, Danielle Tatum, Arthur J. Lustig

**Affiliations:** Department of Biochemistry and Molecular Biology, Tulane University Medical Center, New Orleans, Louisiana, United States of America; Tulane University Health Sciences Center, United States of America

## Abstract

The function of the replication clamp loaders in the semi-conservative telomere replication and their relationship to telomerase- and recombination mechanisms of telomere addition remains ambiguous. We have investigated the variant clamp loader Ctf18 RFC (Replication Factor C). To understand the role of Ctf18 at the telomere, we first investigated genetic interactions after loss of Ctf18 and TLC1 (the yeast telomerase RNA). We find that the *tlc1▵ ctf18▵* double mutant confers a rapid >1000-fold decrease in viability. The rate of loss was similar to the kinetics of cell death in *rad52▵ tlc1▵* cells. However, the Ctf18 pathway is distinct from Rad52, required for the repair of DSBs, as demonstrated by the synthetic lethality of *rad52▵ tlc1▵ ctf18▵* triple mutants. These data suggest that each mutant elicits non-redundant defects acting on the same substrate. Second, interactions of the yeast hyper-recombinational mutant, *mre11A470T, with ctf18▵* confer a synergistic cold sensitivity. The phenotype of these double mutants ultimately results in telomere loss and the generation of recombinational survivors. We observed a similar synergism between single mutants that led to hypersensitivity to the DNA alkylating agent, methane methyl sulphonate (MMS), the replication fork inhibitor hydroxyurea (HU), and to a failure to separate telomeres of sister chromatids. Hence, *ctf18▵* and *mre11A470T* act in different pathways on telomere substrates for multiple phenotypes. The *mre11A470T* cells also displayed a DNA damage response (DDR) at 15°C but not at 30°C while *ctf18▵* mutants conferred a constitutive DDR activity. Both the 15°C DDR pattern and growth rate were reversible at 30°C and displayed telomerase activity *in vivo*. We hypothesize that Ctf18 confers protection against stalling and/or breaks at the replication fork in cells that either lack, or are compromised for, telomerase activity. This Ctf18-based function is likely to contribute another level to telomere size homeostasis.

## Introduction

Telomeres, the nucleic acid/protein complexes present at the termini of eukaryotic chromosomes, provide a means for both end replication and end protection [1]. The primary activity that compensates for replicative attrition of telomeric sequences is the ribonucleoprotein telomerase that catalyzes the addition of simple sequence G+T tracts onto pre-existing telomere sequences. Pathways of recombinational lengthening occur less frequently, normally when under selection for genome stability. In addition, a multiplicity of specific end binding, recombinational, and repair factors act at the telomere. Although these processes have been intensively studied, less is known about the integration of semi-conservative replication in telomere stability and telomere tract size homeostasis.

Ctf18 was first identified in a screen for mutations in chromosome transmission fidelity [2]. *CTF18* encodes one of three variants of the Replication Factor C (RFC) large subunit Rfc1: Ctf18, Elg1, and Rad24 [3], [4]. Each of the variant complexes performs both specialized and redundant roles in PCNA-dependent and, in some cases, -independent clamp loading and unloading. The Ctf18/Dcc1/Cdf8 complex of the variant Ctf18 RFC assists in the loading and in the passage of the replication fork through the PCNA clamp [3].

The loss of Ctf18 is associated with numerous defects [2], [5], [6] including a) the failure to maintain replication rates, b) the inability to repair or restart stalled replication forks, and c) the conversion of replication-induced DNA damage into double strand breaks (DSBs) for DSB repair [5]. *ctf18▵* mutations also confer defects in telomere size regulation [7], localization of telomeres to the nuclear membrane [7], and association with putative telomere-related proteins (e.g., Mdt1) [8]. Ctf18 may also be involved in the S-phase-specific telomeric associations that initiate sister chromatid cohesion (SCC). SCC is extended and maintained during M phase, with separation occurring only at anaphase[9].

Cells containing DSBs and single-stranded DNA exhibit a DNA Damage Response (DDR) through the Tel1 (yATM) and Mec1 (yATR) checkpoint pathways, respectively. In the Tel1 pathway, the Mre11/Rad50/Xrs2 (MRX) complex acts as a sensor for blunt-ended DNA breaks. MRX also acts as part of a downstream helicase/nuclease resection complex [10], [11]. In this context, the telomere can be considered as a unique double strand break [12]. Overall, the Mre11 complex plays multiple key functions in homologous recombination, in non-homologous end joining, and in telomere size homeostasis. Several comprehensive reviews have focused on these roles of Mre11 in both yeast and vertebrates [10], [13].

Telomere size homeostasis has been directly observed in both yeast and human cells [14]–[17]. A paradigm has emerged in yeast in which Tel1 and Mre11 bind preferentially to short telomeres [18] and are removed by Rif1 and Rif2 at long telomeres [19]–[22]. The subsequent processing of the shorter terminus with other nucleases and helicases [23]–[26], converts the telomere into a 3’ terminal single-stranded overhang. This change in structure and the cell-cycle dependent phosphorylation of Cdc13 (yCdt1) by Cdk1 leads to association and activation of telomerase at the 3’ single-stranded overhang [27].

The MRX complex is also required for one class of recombinational telomere elongation in telomerase-negative cells, termed Type II recombination [28]–[30]. An MR complex is also required for telomeric rapid deletion (TRD) [31], [32], an intra-chromatid recombination event that deletes elongated telomeres to wild type size . Recombinational telomere elongation (RTE) can compensate for this loss, which is similar to the vertebrate alternative (ALT) pathway [33], [34].

As part of a screen to identify mutations that decrease TRD rates [31], we identified and characterized a mutation (*mre11A470T*) that lies within a highly conserved motif in the α helical coiled/coil region of Mre11 [35], [36].Interestingly, X-ray crystallography has shown that this coiled-coiled site is part of an Mre11 association site with Rad50 [36], [37]. Our previous studies of this mutant revealed several major phenotypes including: a) the absence of senescent growth in telomerase-negative cells, b) an apparent increase in telomeric BIR in telomerase-negative cells; and c) an inability of telomerase to utilize “seed sequences” for telomere elongation, suggesting constraints on telomerase action [35].

In this study, we find that the growth of telomerase-negative cells is dependent on the clamp loader variant Ctf18 RFC. Curiously, this dependency differs from replicative telomere attrition and double strand break repair, most likely due to an early increase in a failure to repair DNA damage and to increased stalling and/or breaking at the replication fork.

The *mre11A470T ctf18▵* double mutant confers cold sensitivity and several additional key phenotypes including telomere shortening, hypersensitivity to both hydroxyurea (HU) and methane methyl sulphonate (MMS), DNA Damage Response (DDR) induction, and a failure to separate the telomeres of sister chromatids. Survivors of cold sensitivity can be restored to the *mre11A470T ctf18▵* phenotype through a shift to growth at the permissive temperature (30°C). During this shift to 30°C, telomerase can re-elongate telomeres. These data suggest that the Mre11/Rad50 junction is critical for regulating telomerase and/or the substrates for telomerase and that the Ctf18 RFC is required for maintaining telomeric replication.

## Materials and Methods

We do no animal or human studies. Our studies are restricted to the yeast *Saccharomyces cerevisiae*.

### Media

All strains used in this study were n isogenic to the W303 background. Cells were grown at the indicated temperatures in YPD (1% yeast extract, 2% peptone, and 2% glucose) or YPAD (YPD + 6 mg/ml adenine) media at 15°C or 30°C. When centromere (*CEN*) plasmids were present in yeast strains, growth was under selection for a plasmid marker gene on synthetic omission media or media containing G418 to assay kanamycin resistance.

### Strains

A non-isogenic strain containing the *ctf18▵* allele, disrupted by a kanamycin resistance cassette (Open Biosystems), was initially generated as part of the yeast deletion library consortium. The disruption allele was amplified by PCR and used to replace the *CTF18* gene in W303 (kind gift of Rodney Rothstein; also termed wild type in these studies). The *mre11A470T*-containing strain, KMM4, was isolated in our laboratory as described [35]. The *mre11A470T ctf18▵* double mutant was generated in this study by standard methods at 30°C. All candidate deletion strains used in this study were obtained from the kanamycin deletion project through Open Biosystems. The *CTF18*-containing plasmid p5472 was kindly provided by Phil Heiter. All other strains were developed in our lab or obtained through multiple collegial exchanges.

The telomere-proximal *lacO* arrays were constructed by crossing the strain YLA1119 (*Mata*, isogenic to W303; a kind gift of Robert Skibbens), carrying both a telomeric array of 275 *lacO* binding sites for LacI (Douglas Koshland, personal communication) and the LacI-GFP fusion protein [9], [38] to W303 as well as isogenic strains carrying the *mre11A470T*, *ctf18▵* or *mre11A470T ctf18▵* alleles. The resultant diploids were sporulated and haploid spore colonies carrying each respective *mre11 or ctf18* allele (as well as the *lacO* array and the plasmid-encoded LacI-GFP) were transformed with *TUB1*-GFP fusion protein on *URA3*-containing *CEN* plasmid, giving rise to HG109, HG110, HG111 and HG112. These strains contained the *MRE11CTF18*, *mre11A470T CTF18*, *MRE11ctf18▵* and *mre11A470T ctf18▵* alleles, respectively.

### Subculturing of CTF18/ctf18▵ TLC1/tlc1▵ and CTF18/ctf18▵ MRE11/mre11A470T spore products

To examine spore viability at the earliest possible time after germination, a strain heterozygous for both *TLC1* and *CTF18* (*TLC1/tlc1::LEU2*) was sporulated and five complete tetrads were evaluated after dissection in a double blind study. At regular intervals, the spores were examined microscopically and notations made of their growth phenotypes. We examined spores for very slow growing colonies, colonies that arrested transiently after forming irregular micro-colonies, and spores that germinated, but underwent only a few cell divisions. The growth behavior was monitored at one-day intervals after germination at 25°C. The genotype of each strain was determined after sufficient growth to assay markers allowing us to determine or surmise the segregation pattern of each spore colony.

For liquid subculturing, wild type, *tlc1▵*, *ctf18▵*, and *tlc1▵ ctf18▵* cells were grown for 18 hours in YPD at 30°C, and subsequently stored at 6 hours at 4°C. Cells were diluted to 1×10^5^ cells/ml in 5 ml of fresh YPD and grown for another 18 hours at 30°C (36 hours). The subculturing was repeated for eight additional periods of 18 hours of growth until 198 hours of subculturing following germination (100 wild type generations) was reached. Cells were analyzed after each 18 hours of growth for cell density, using a hemocytometer, and for viability, as assayed by the decrease in the fraction of cells in each subculture capable of continued growth following plating of cell dilutions.

Subculturing of *rad52▵ tlc1▵* cells was conducted for several rounds of 18 hours each. DNA was isolated from the initial subcultures if sufficient cell density was present. Liquid subculturing of the spores derived from the *CTF18/ctf18▵ MRE11/mre11A470T* diploid was conducted under identical conditions at 30°C, except that viability was measured only on alternating subcultures. No change in growth was observed over multiple liquid subcultures of the same strain grown at 30°C.

For solid subculturing, a single colony of *mre11-A470T ctf18Δ* grown at 30°C was used to seed cells onto plates shifted to 15°C, and grown for 5 days at a temperature of 15°C before cells were collected and plated again onto YPAD plates. After two rounds of sub-culturing at 15°C, *mre11A470T ctf18Δ* cells ceased to grow. Rare cold resistant colonies arose after 10 days of incubation at 15°C.

### Identification and Characterization of Survivors

Survivors of the liquid growth of *tlc1▵* and *tlc1▵ ctf18▵* double mutants after 198 hours of culturing were subcloned and individual colonies were identified. DNA was isolated from multiple individual subclones and telomere lengths determined by Southern analysis.

To assay for the reversibility of cold sensitivity, three individual survivor clones were shifted from 15°C to 30°C for up to five cycles of continual liquid subculturing. Telomere lengths of the DNAs were characterized by Southern analysis. As a control, growth and viability were assayed in strains carrying each spore genotype (generated from *CTF18/ctf18▵ MRE11/mre11A470T* diploids) after 198 hours of growth following germination in YPD at 30°C.

### Synthetic lethality of *rad52▵ ctf18▵* strains in the absence of *TLC1*

A diploid strain, CP1 (TLC1/*tcl1*::*LEU2* CTF18/*ctf18*::KanMX (kan^r^) RAD52/*rad52*::*TRP1)* was constructed by consecutive one-step gene disruptions of one of the two diploid homologs using PCR fragments of *tcl1*::*LEU2*, *ctf18*::*KanMX*, and *rad52::TRP1*. Gene disruptions were confirmed by marker tests and PCR assays of each locus. CP1 was transformed with a *RAD52*-containing *HIS3*/*CEN* vector, pWJ1213 (a gift from R. Rothstein), to generate CP2. Triple mutant spores carrying pWJ1213 were obtained after standard random spore analysis (termed CP3). Diploids carrying the pWJ1213 plasmid were denoted as CP4. The identity of CP3 was confirmed by both mating type and histidine prototrophy. CP3 and CP4 were subsequently grown on YPD for 72 hours to identify colonies without the pWJ1213 plasmid. Following growth, haploids and diploids were determined by mating type, and both were plated onto histidine omission media to score for the presence of the plasmid.

### Telomere Southern Blot Analysis

Telomere Southern blots were carried out as previously described [39], using polyGT/polyCA (poly GT) as the hybridization probe. The signals from the blot were detected using the Typhoon Phosphorimager (GE) as well as by multiple film exposures. In some cases, the blots were stripped and probed using *PEP4* sequences as loading controls.

To determine the size of small molecular weight fragments, Image J software was used to superimpose the 1 kb DNA ladder from the Ethidium Bromide (EtBr) image onto the Southern blot image. The composite image was imported into Total Lab and the sizes of the control ladder fragments were used to create a regression curve of pixel position and molecular weight. Regression analysis provided an equation that was then used in combination with the pixel positions of the telomere bands to estimate the molecular weight (in kb).

### Protein Extract Preparations

Yeast cellular protein extracts were prepared by a modification of the trichloroacetic acid [40] method. Briefly, 4×10^8^ cells were collected by centrifugation and washed with deionized water and 500 µl of 20% TCA. The pellet was re-suspended in 200 µl of 20% TCA. Cells were lysed by vortexing with glass beads for 30 minutes at 4°C with intermittent cooling on ice. The supernatant was transferred to a new 1.5-ml micro-centrifuge tube and the beads were washed twice with 200 µl of 5% TCA. The supernatants were pooled and centrifuged for 10 minutes at 3,000 rpm at 4°C.

### Rad53 Phosphorylation Analysis

Cultures were grown at 30°C in YPD to late logarithmic phase (A_600_ ∼1.0) and collected by centrifugation. The protein pellets, isolated as described above, were dissolved in 1X sodium dodecyl sulfate-polyacrylamide gel electrophoresis (SDS-PAGE) loading buffer, supplemented with both 20 µl of 1 M dithiothreitol (DTT) and 100 µl of 1 M Tris base (pH 9). The samples were boiled for 3 minutes and centrifuged at 14,000-x *g* for 5 minutes. Proteins in the whole cell extracts were resolved on a 10% SDS-PAGE gel and transferred onto a polyvinylidene difluoride (PVDF) membrane (Immobilon-P; Millipore) via a Bio-Rad tank transfer system. Rad53 was probed with goat anti-rabbit Rad53 (1∶1000; Santa Cruz), which reacts with both phosphorylated and non-phosphorylated forms of Rad53, followed by incubation with goat IgG-HRP (1∶5000; Santa Cruz). Blots were incubated with the ECL Prime Western Blotting Detection Reagent (Amersham), and the protein species were detected using either a Bio-Rad chemiluminescence scanner (VersaDoc Imaging System) or by autoradiography. Identical experiments were performed for cold sensitive survivors before and after shifting to 30°C.

### Sister Telomere Separation

HG109, HG110, HG111 and HG112 were grown asynchronously at either 15°C or 30°C overnight in YPD liquid media. 100 anaphase cells were identified morphologically at each temperature and the separation of sister chromatid telomeres was documented in anaphase cells. The labeling pattern was determined by photography using a Nikon 80i fluorescence microscope equipped with a 100x objective.

In all cells, telomeres were labeled with the LacI-Green Fluorescent Protein (GFP) fusion protein and with *TUB1*-GFP (tubulin-GFP; Cell Signaling) for the labeling of microtubules. The microtubules provide a location marker to more easily locate and visualize the telomere signals. Bright field images of the same cells were also generated. We were unable to use red fluorescence protein (RFP) for tubulin staining due to rapid bleaching of the fluorophore. Telomeres could be observed as discrete signals while microtubules were more diffuse in control cells while they were more disorganized in mutants lacking telomere separation.

### Measuring Telomerase Activity In Vivo

A mutated version of the telomerase template RNA *TLC1*, *TLC1-1,* incorporates HaeIII sites into the telomere [41] was cloned into a *URA3-*containing integrating plasmid. The wild-type TLC1 plasmid was integrated at the genomic *TLC1* locus by transformation into both *MRE11 CTF18* wild type strains and three independent cold sensitive survivors of *mre11A470T ctf18▵* after shifting to the permissive temperature of 30°C. As a result, duplications were created consisting of one copy each of *TLC1* and *TLC1-1* in all strains. Wild type *MRE11 CTF18* strains have a slow turnover of terminal sequences under homeostatic conditions, and were used as a negative control. To visualize the presence of telomere HaeIII sites, the DNA was treated with two restriction enzymes (AluI and MboI) that have 4 bp recognition sites in order to reduce the size of most genomic non-telomeric fragments. One half of the DNA digest was then treated with HaeIII and a Southern analysis of the DNA was performed using poly GT as a probe.

## Results

### A Screen to Examine Potential Synthetic Interactions with *mre11A470T*

Our initial screen was intended to identify genes encoding proteins, that, when lost, require a function of Mre11. Such associations may be hidden in the face of multiple interactions, or may vary in severity in other *mre11* alleles. Among the candidates, we identified null alleles of *MDT1*, *MSH2*, *CLB2*, *UBP2* and *CTF18*, and analyzed them in combination with the *mre11A470T* mutation under a variety of conditions, including cold sensitivity at 15°C. Only the *mre11A470T ctf18▵* double mutant conferred an almost complete lack of growth at 15°C. Interestingly, cold sensitivity has previously been associated with telomere entanglements [42]. This double mutant also conferred a broad range of other conditional telomere-related genetic and cytological phenotypes. We therefore focused exclusively on the function of the RFC subunit 1 variant, Ctf18, at telomeres.

### Rapid Senescence in *tlc1▵ ctf18▵* Cells

To ascertain a telomeric role of Ctf18 in replication stalling and chromosome breakage**,** we tested the effect of loss of Ctf18 in a telomerase-negative system. To probe for any potential early-arising double mutant phenotypes, we compared the rate of growth of *CTF18 tlc1▵* cells lacking the telomerase RNA subunit, and the *tlc1▵ ctf18▵* double mutants at identical times after germination of 5 tetrads dissected from doubly heterozygous strains. A significant number of spores displayed periods of arrested growth as well as termination of growth after several generations of growth. These deficiencies, however, were not linked to the *tlc1▵ ctf18▵* double mutant. Rather, they were likely to be the consequence of the known haplo-insufficiencies of both the *TLC1 and CTF18* genes [43], [44].

**Figure 1:**
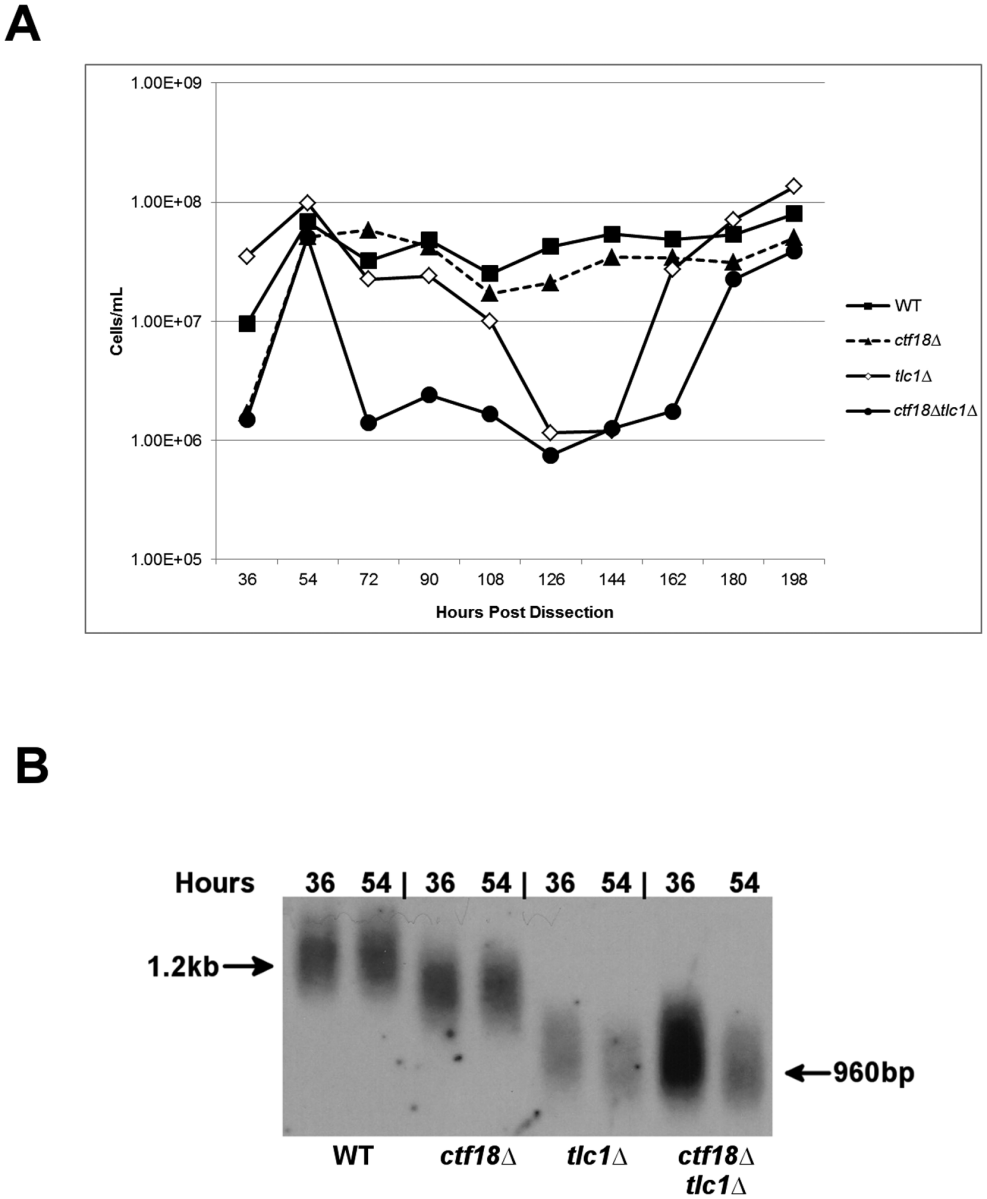
Senescence of telomerase-negative strains in the presence and absence of Ctf18. [**A**]: Senescence assay growth curve generated during 196 (11 rounds) of repetitive subculturing for wild type, *mre11A470Ttlc1▵*, *ctf18▵* and *mre11A470T ctf18▵* strains. Colonies from freshly dissected spores were incubated on YPD plates for 18 hours at 30°C and then inoculated into 5 ml YPD, grown at 30°C for 18 hours [36 hours after dissection], and counted using a hemocytometer. Each subsequent round of subculturing [hours 54–198] was initiated by inoculating fresh medium with 1×10^5^ cells/ml from the previous subculturing at 30°C. [**B**]: Telomere lengths of the strains in [1A] after 36 and 54 hours of growth after dissection at 30°C. The samples shown on the gel were derived from the cells grown in [**1A**]. Telomere sizes were determined by digestion of genomic DNA with XhoI followed by Southern analysis using poly GT as a telomeric probe.

Significant phenotypic changes that were specific to the double mutant were observed after 54 hours of subculturing after germination. Although cells grew to wild type densities (**Figure 1A**), telomere size distributions were qualitatively similar in both *tlc1▵* and *tlc1▵ ctf18▵* cells, displaying telomere lengths that were approximately 200 bp shorter than the 300 bp found in wild-type strains (**Figure 1B**). Hence, changes in growth in the *tlc1▵ ctf18▵* mutants relative to the *tlc1▵* mutant were present and were not the consequence of short telomere size alone. These data suggest the presence of some form of defect at the replication fork (although lower than the sensitivity of two-dimensional gel electrophoresis, data not shown). Furthermore, the fraction of cells in each subculture that was capable of continued growth (operationally termed viability) was between 10^−3^ and 10^−4^ of *tlc1▵* cell density (**Figure S1**).

One possible explanation for an increase in the loss of viability could be an inability to repair DSBs. Misprocessing of DSB repair has been previously observed in *ctf18▵* mutants [5]. To test if such a defect produced any small fragments, we first tried to identify telomere-hybridizing products of uncut *tlc1▵ ctf18▵* DNA after agarose gel electrophoresis and Southern analysis using poly GT as a probe (data not shown). No truncated products were found, although the level of detection could be compromised by a diffuse distribution.

**Figure 2:**
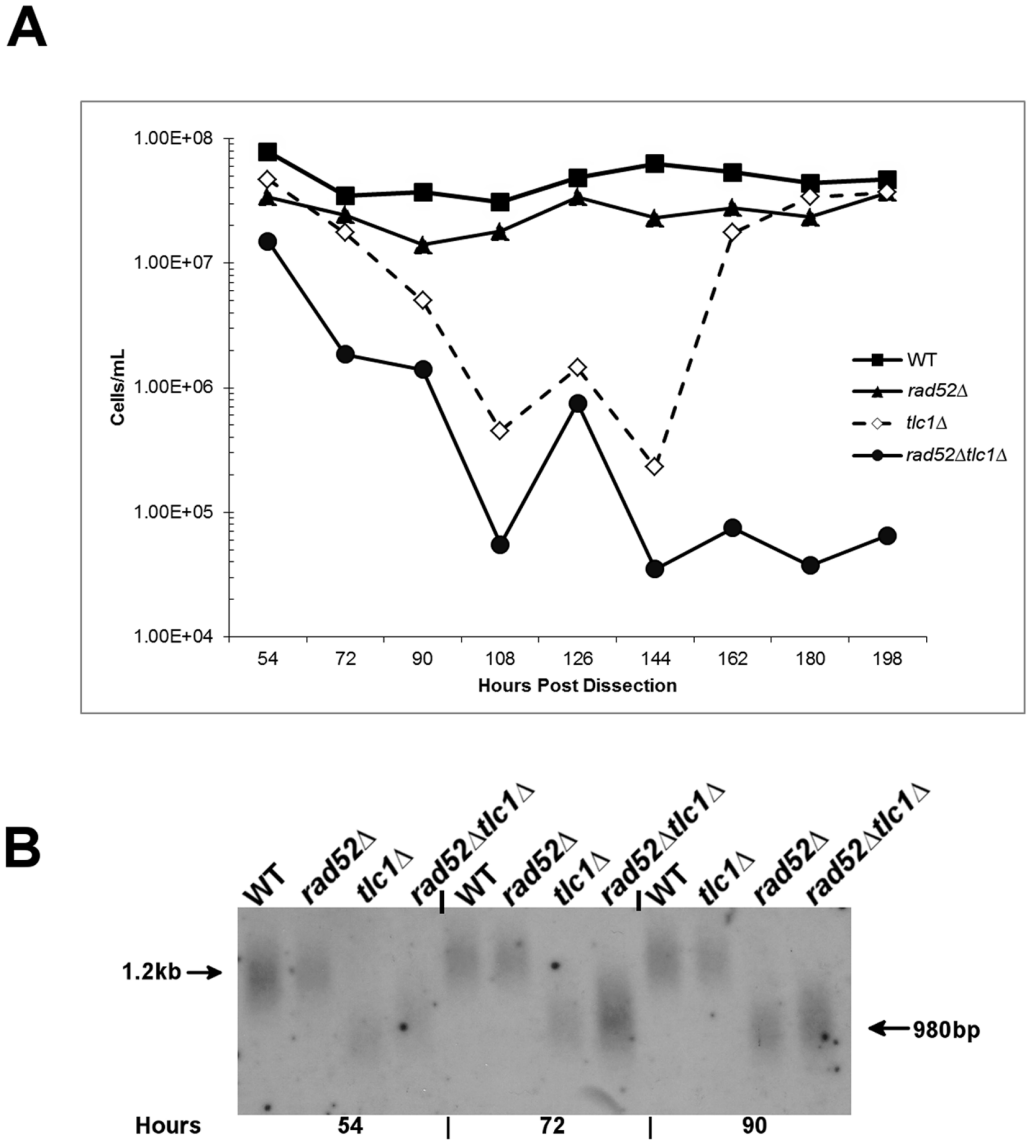
Senescence of telomerase-negative strains in the presence and absence of Rad52. [**A**]: Cells were subcultured for eleven rounds (198 generations post germination) of subculturing for the indicated strains as described above. Colonies from freshly dissected spores were incubated on YPD plates for 36 hours at 30°C, and 1×10^5^ cells were inoculated into 5 ml of YPD at 30°C for 18 hours [54 generations after dissection], and cells counted using a hemocytometer. Each subsequent 18-hour round of grown [hours 72–198] was initiated by inoculating 1×10^5^ cells into fresh YPD. [**B**]: Telomere lengths of the indicated strains in [**2A**] from hours 54, 72 and 90 hours after dissection were determined by Southern analysis of XhoI digested DNA using poly GT as a probe.

Second, previous investigations and findings in this study indicate that *tlc1▵ rad52▵* confers a severe loss in cell growth compared to *tlc1▵* cells [30] (**Figure 2**). We tested whether *rad52▵ tlc1▵* generated shorter telomeres relative to *tlc1▵.* Such a loss of growth could be due to a failure of Rad52 to repair telomeric double strand breaks. However, although telomere sequence was clearly lost in *tlc1▵ rad52▵* cells, the loss was not more extreme than for cells carrying the *tlc1▵* mutation alone, inconsistent with this hypothesis.

The third possibility is that *ctf18▵* and *rad52▵* mutations lie in the same pathway in telomerase negative cells. If the acceleration of cell death or arrest were acting through the same pathway in *tlc1▵ ctf18▵* and *tlc1▵ rad52▵ cells*, the triple mutant should not differ significantly in viability or telomere structure from *tlc1▵ rad52▵* double mutants. If however, *tlc1▵ ctf18▵* low growth rates were due to a distinct mechanism of telomere loss, the absence of Rad52 should result in a synergistic decrease in viability.

**Figure 3:**
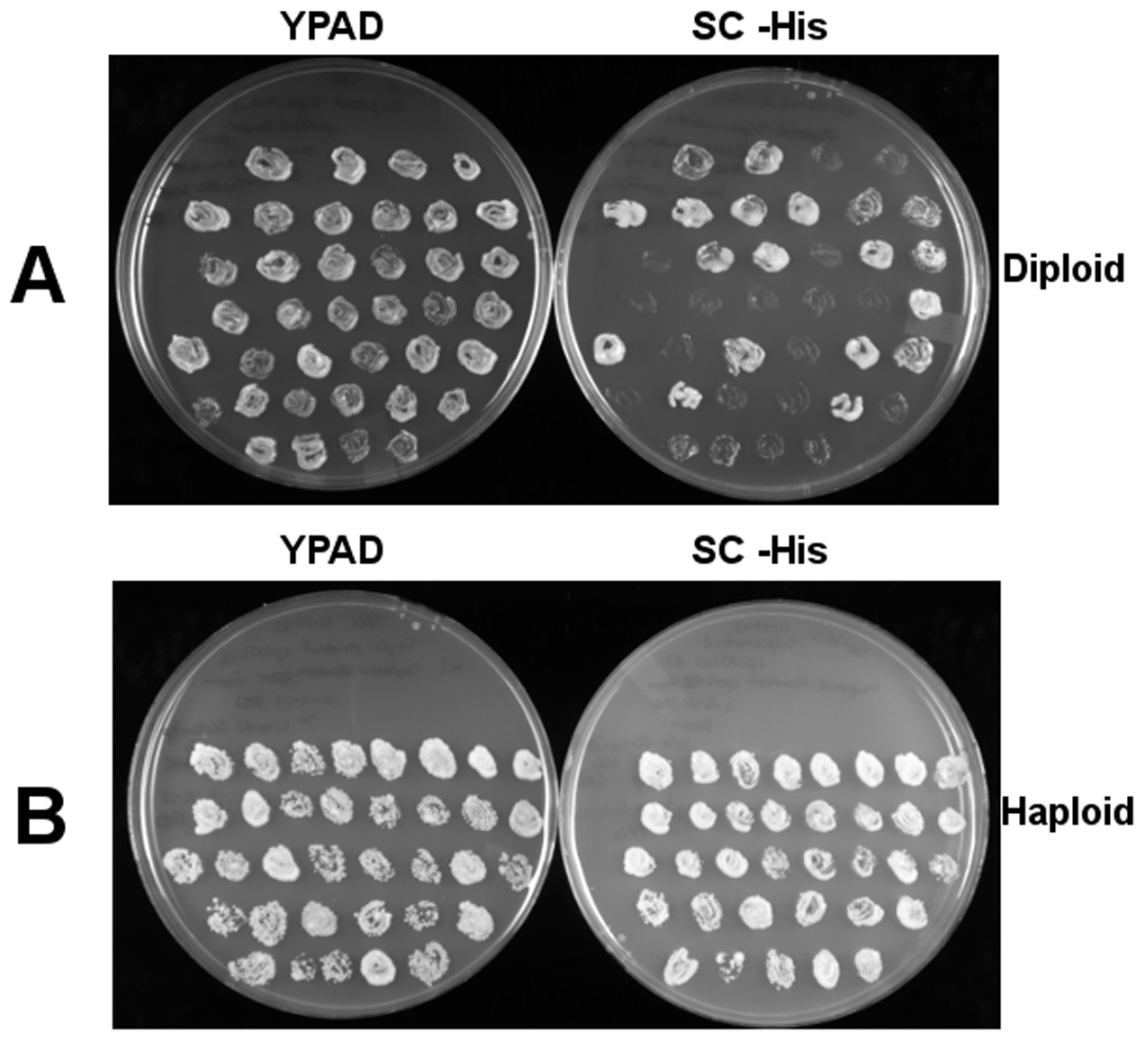
*rad52▵ tlc1▵ ctf18▵* haploid strain requires *RAD52* for viability. [**A**]: The CP1 diploid heterozygous for *tcl1*::*LEU2*, *ctf18*::*KanMX*, and *rad52*::*TRP1* were transformed with the *RAD52*-containing *CEN* plasmid, pWJ1213, giving rise to CP2. CP2 cells were grown on solid YPD medium for 72 hours to allow for plasmid loss. Single colonies were then patched onto both histidine-omission media and YPD to determine the rate of plasmid loss. [**B**]: Products of random spore analysis of CP2 grown on YPD for 72 hours in an attempt to isolate a viable haploid *tcl1*::*LEU2*, *ctf18*::*KanMX*, and *rad52*::*TRP1* strain. Of the 75 triple mutant haploid spores tested, none were able to lose the plasmid, as evidenced by continued growth on histidine-omission media, and retention of growth on YPD.

To distinguish between these two possibilities, diploids heterozygous for all three markers were transformed with a *RAD52*/*HIS3*/*CEN* plasmid. Following sporulation, random spores were then sought that would carry all of the mutations, with *rad52▵* being complemented by the plasmid-borne wild-type *RAD52*. Cells were then grown on YPD, and a mating type test was used to distinguish haploids from diploids. Diploid cells lost the plasmid at a rate of 30% (**Figure 3** **, top**). In contrast, out of 75 colonies, the *RAD52*/*HIS3*/*CEN* plasmid was never lost in haploid cells after growth on YPD. These data suggest that the triple mutant is either synthetically lethal or has a growth defect far more severe than either double mutant (**Figure 3** **, bottom**). We conclude that the two pathways act synergistically in the loss of viability in telomerase-negative cells.

### Survivors of *tlc1▵* and *tlc1▵ ctf18▵* Mutant Cells

In liquid culture, *tlc1▵* cells start producing survivors at the fifth round of subculturing (116 hours), while *tlc1▵ ctf18▵* produce survivors at the eighth round of subculturing (**Figure S2**). DNA was isolated from multiple subclones at the fifth round and eighth round of subculturing for the *tlc1▵* and *tlc1▵ ctf18▵* mutants, respectively, subjected to Southern analysis. Most survivors of *tlc1▵* mutants under liquid conditions produced multiple large fragments of discrete telomere size [29]. There are subtle, but significant, differences in the *tlc1▵ ctf18▵* late-arising survivors, with double mutants displaying a larger population of similarly sized elongated Type II telomeres, as well as the apparent presence of Type I telomeres. The mechanistic basis for differences between *tlc1▵* and *tlc1▵ ctf18▵* is under investigation. However, larger telomeres were most prevalent among survivors, suggesting that the double mutant regains the initial growth rate of *tlc1▵ ctf18▵* cells. These data indicate that, at least in large part, through the direct action on the telomere.

### A Genetic Interaction Between the Mre11/Rad50 Interface and the Ctf18 RFC Clamp Loader

**Figure 4:**
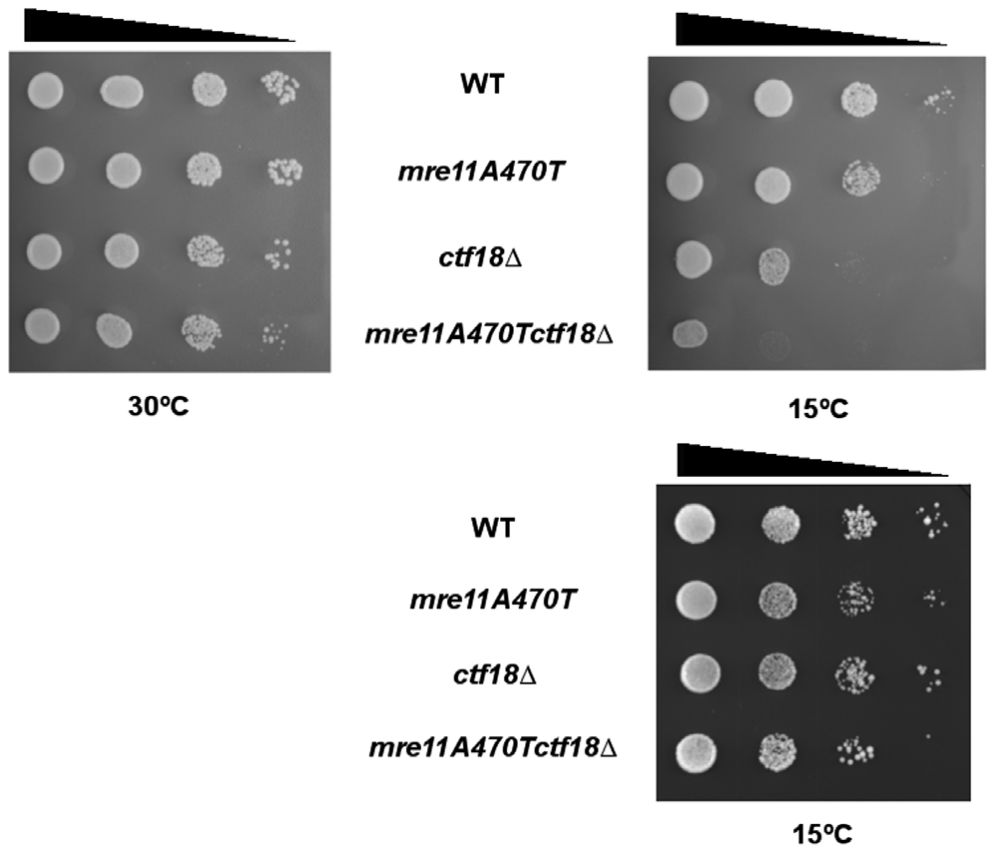
*mre11A470T ctf18Δ* is cold sensitive for growth. [Top] Ten microliters of wild type, *mre11A470T*, *ctf18▵* and *mre11A470T ctf18▵* cultures grown at 30°C were spotted onto YPD plates at 10-fold serial dilutions [arrow] and incubated at either 30°C [left] for two days or at 15°C [right] for five days to compensate for the lower growth rate of the latter. [Bottom] Cells of the indicated strains were also transformed with the *CTF18*-containing plasmid p5472 were spotted onto uracil omission media at 10-fold serial dilutions. Plates were then incubated at 15°C for fourteen days.

Multiple investigations have revealed an association between cold sensitivity and telomere entanglements [45], [46]. We therefore test growth at 15°C and 30°C in wild type, *mre11A470T*, *ctf18▵* and *mre11A470T ctf18▵* strains. The *mre11A470T ctf18*▵ double mutant grew well at 30°C, but lost viability when grown at 15°C. Partial cold sensitivity was also conferred by the *ctf18▵* mutation. The cold sensitivity was complemented by the introduction of *CTF18/CEN*, as expected (**Figure 4** **)**.

**Figure 5:**
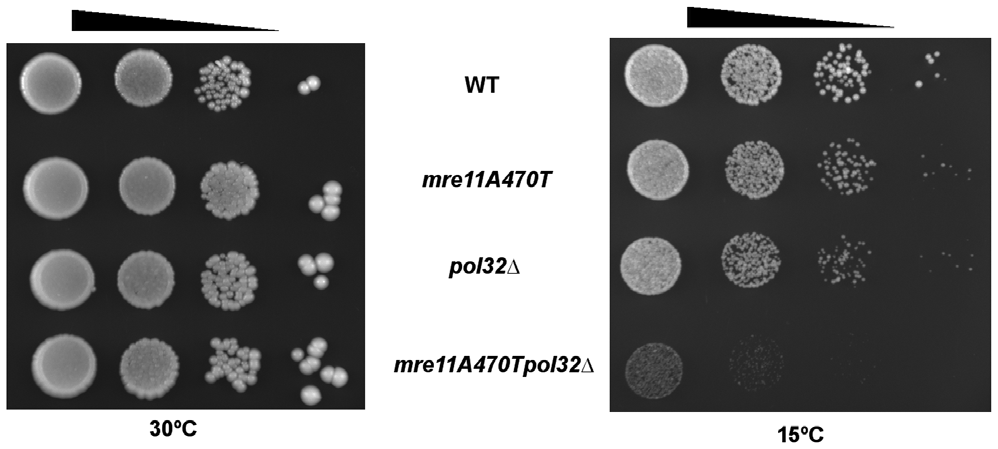
*mre11A470T pol32 Δ* is cold sensitive for growth. Ten microliters of wild type, *mre11A470T*, *pol32▵* and *mre11A470T pol32▵* cultures grown at 30°C were spotted onto YPD plates at 10-fold serial dilutions [arrow above] and incubated at either 30°C [left] for two days or at 15°C [right] for five days of growth.

As Ctf18 is a component of a PCNA clamp loader, we hypothesized that a mutation in another PCNA (Pol30)-associating protein may influence the same pathway. One candidate protein is Pol32, a subunit of DNA polymerase ∂ that is required for error-prone DNA synthesis. Pol32 also interacts with PCNA in both physical and genetic assays [47]. We therefore tested the growth properties of single and double mutants. While no cold sensitivity was observed in the *pol32▵* allele alone, *pol32▵ mre11A470T* cells displayed a cold sensitive profile similar to the *mre11A470T ctf18▵* mutant (**Figure 5** **)**. Hence, it is likely that Ctf18 and Pol32 are operational components of the same pathway in PCNA formation and/or function.

### *mre11A470T ctf18▵* Defects in Replication

**Figure 6:**
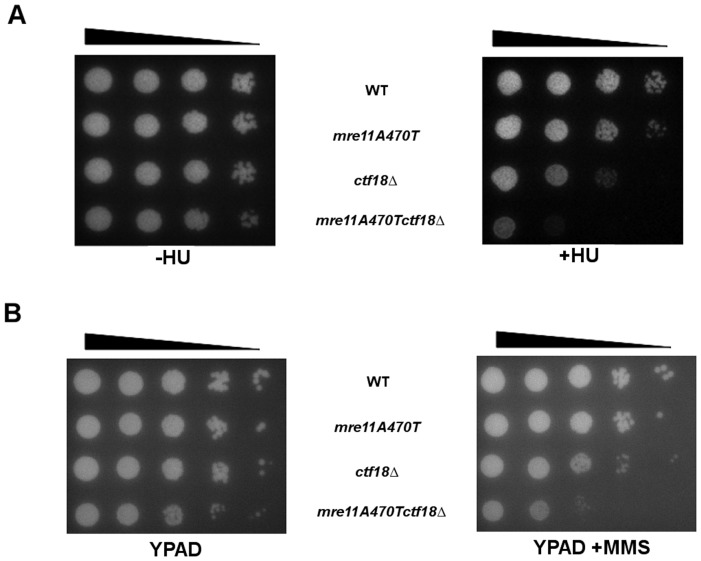
*mre11A470T ctf18Δ* cells are sensitive to replicative inhibitors and alkylating mutations. Wild type, *mre11A470T*, *ctf18Δ* and *mre11A470T ctf18Δ* cultures grown at 30°C were spotted onto YPAD in 10-fold serial dilutions [arrow on top] without [left] or with [right] 40 mM of the replicative inhibitor hydroxyurea [HU] and incubated at 30°C. The same cells were treated in the presence or absence of 0.0005% methane methyl sulphonate (MMS) and incubated at 30°C.

**A) Sensitivity to Replicative DNA Damage Agents.** *mre11A470T ctf18▵* double mutants exhibit a synergistic >10^5^-fold increase in sensitivity to two different forms of replication stress. First, the ribonucleotide reductase inhibitor hydroxyurea (HU) [48] terminates replication due to the lowered concentrations of dNTP. Similarly, the double mutant is extremely sensitive (relative to single mutants) to the alkylating agent MMS, that mutagenizes replicating DNA ultimately leading to checkpoint arrest (**Figure 6**). These synergistic defects suggest that the Rad50/Mre11 interface and Ctf18 play non-redundant functions at the replication fork.

**B) DNA Damage Response (DDR).** Mre11 acts both as an upstream regulator of the ATM pathway and as an ATM-stimulated enzymatic activity that facilitates the formation of resected DNA fragments, in the presence of additional rescission enzymes [23]–[26]. In most cases, the ATM pathway responds to DSBs with minimal single stranded regions, while the ATR pathway responds to the nicks, gaps and other forms of single stranded DNA. In both ATM and ATR pathways, DDR can be measured by the phosphorylation of the highly conserved Rad53 [yChk1] protein [49], resulting in a cascade of various recombination, nucleolytic and repair enzymes necessary to complete the checkpoint-mediated repair [10].

**Figure 7:**
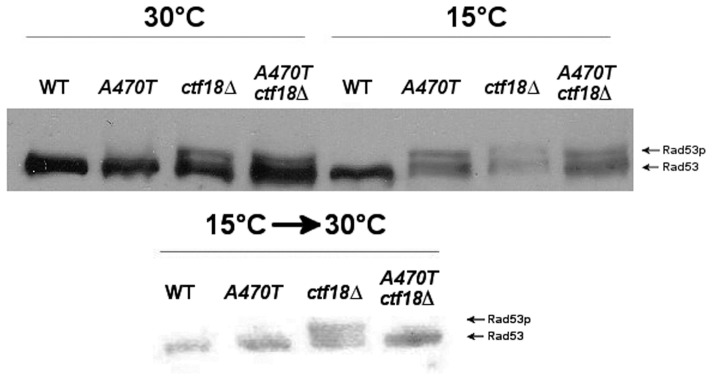
Western blot analysis of DNA damage responses to *mre11A470T* and *ctf18*▵ Mutations. [**Top**]: Protein extracts were isolated from wild-type, *mre11A470T*, *ctf18▵* and *mre11A470T ctf18▵* cultures grown in 5 ml YPD cultures at 30°C or 15°C. Extracts were subjected to electrophoresis on a 10% SDS-PAGE gel prior to Western analysis using goat primary polyclonal anti-Rad53 antibody as described in Materials and Methods. Phosphorylated forms (arrows on right) migrate more slowly than the un-phosphorylated species. [**Bottom**]: Protein extracts were isolated from cold resistant *mre11A470T ctf18Δ* survivors and grown in 5 ml YPD at 15°C before shifting to 30°C. Extracts were subjected to Western analysis for Rad53 expression as described above.

Phosphorylation retards the mobility of Rad53 on polyacrylamide-SDS gels as visualized on Western blots. Wild type cells show little sign of Rad53 phosphorylation at 15°C or 30°C, while *ctf18▵* cells phosphorylate Rad53 at both temperatures. In contrast, *mre11A470T* confers phosphorylation exclusively at 15°C. It is conceivable that *mre11A470T*-induced damage at low temperatures may qualitatively contribute to *mre11A470T ctf18▵* cold sensitivity (**Figure 7**).

**C) Telomere Separation at Anaphase in Wild Type,** ***mre11A470T, ctf18▵,*** **and double mutant cells.** Two pathways in yeast contribute to cohesion of sister chromatids. The pathway described here involves the formation of the cohesion complex at the replication fork and serves to initiate sister chromatid cohesion through the formation of ‘sister chromatid telomeres’. In contrast, M phase is responsible for the completion and maintenance of sister chromatid cohesion. Thus, defects in the initiation of cohesion in S phase are not observed until anaphase (e.g., [50]). To test whether telomeric defects mediated through Ctf18 are related to sister telomere cohesion, we introduced GFP-LacI and GFP-tubulin in a strain containing 275 copies of telomeric *lac O sites*. In the presence of GFP-lacI, a punctate signal appears at the telomeres of wild type cells. In contrast, GFP-tubulin labels the microtubules along their length during anaphase as a more diffuse signal. In cells with un-separated telomeres, the tubulin signal is more disorganized.

**Figure 8:**
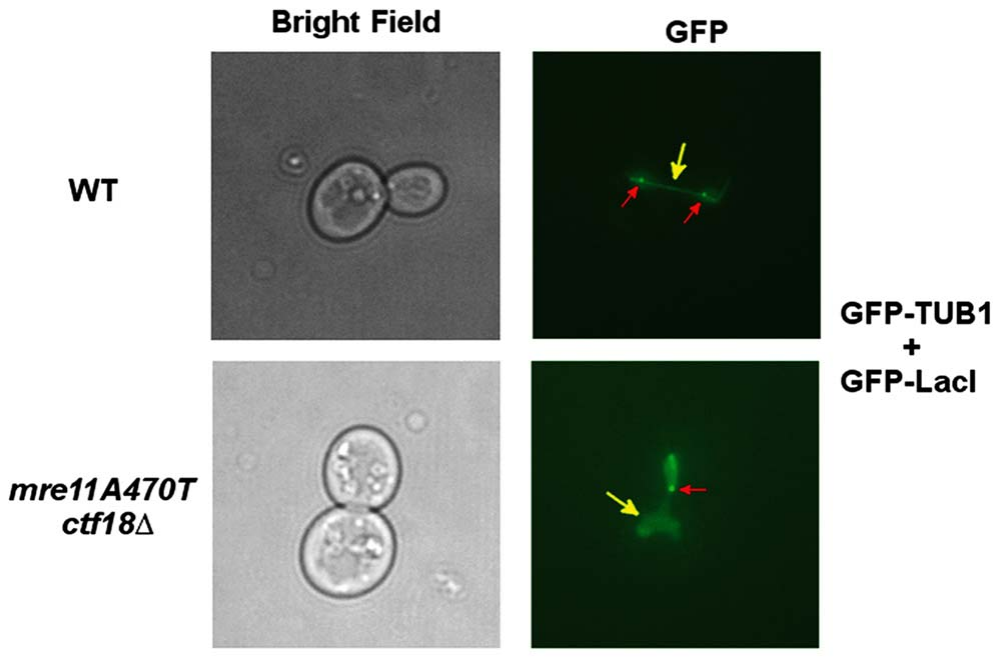
*mre11A470T ctf18Δ* cells have sister telomere separation defects. Late anaphase cells identified from asynchronous cultures of wild-type, and *mre11A470T ctf18▵* strains grown at 30°C and characterized by fluorescence microscopy to visualize GFP signals as described in Materials and Methods. Telomeres on chromosome IV-L were labeled with GFP-LacI fusion proteins bound to LacO arrays adjacent to telomeres. The mitotic spindles were labeled with the *TUB1*-GFP fusion protein. The left panel displays the bright field image while the right panel the fluorescent signal from the same field.

Previous data have shown that defects in *ctf18▵* influences sister chromatid cohesion. We would predict that the loss of cohesion function in S phase is the consequence of defects during replication in *ctf18▵*-containing mutant backgrounds. Analysis of the separation of sister chromatid telomeric *lacO* arrays were conducted at both 15°C and 30°C (**Figure 8**).

**Figure 9:**
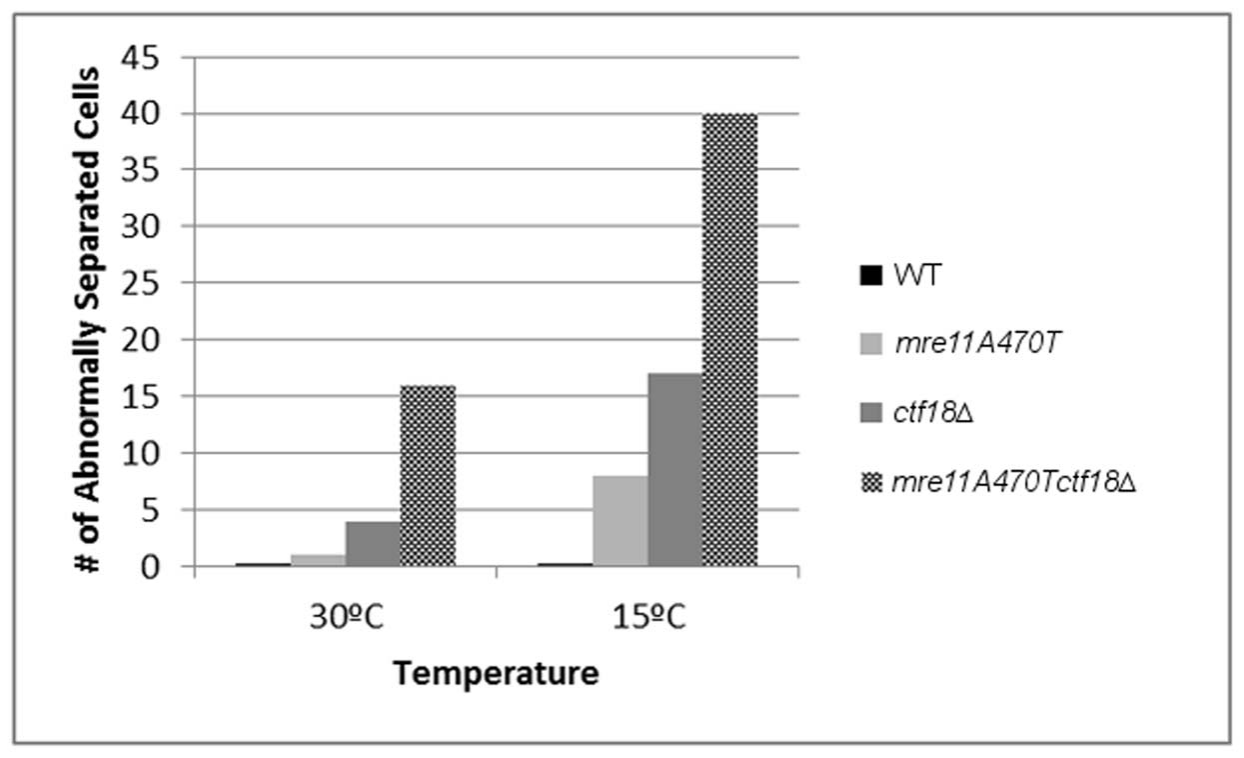
Quantitative analysis of sister telomere separation. 100 late anaphase cells identified after asynchronous growth of wild type (*MRE11*), *mre11A470T*, *ctf18▵*, or *mre11A470T ctf18▵* strains were quantitatively assayed for sister telomere separation as in **Figure 8** **.**

100 anaphase cells were analyzed at each temperature and each strain for as assayed by the loss of punctate spot duplication (operationally termed sister telomere separation in this study) (**Figure** **9**). Morphological abnormalities were present in some cells at 15°C, but not at 30°C. Therefore, we found the 30°C dataset to be more precise. Wild type cells always separated telomeres at 30°C. 1% and 4% of *mre11A470T* and *ctf18▵* cells, respectively, displayed a loss of sister telomere separation. In contrast, 16% of *mre11A470T ctf18▵* failed to separate the telomeres of sister telomeres at anaphase. The effect is more extreme at 15°C, with approximately 60% of *mre11A470T ctf18▵* anaphase cells failing to separate sister chromatid telomeres. The *mre11A470T ctf18▵* cells behaved synergistically with respect to single mutants, indicating the expected (**Figure** **9**). Analogous experiments were conducted in *ctf18▵*, *mre11A470T*, and *mre11A470T ctf18▵* mutant cells. The data is consistent with the presence of two distinct functions acting in the process of sister telomere cohesion.

### Formation of Survivors After Growth at 15°C

Two major classes of RAD52-dependent recombinational survivors have been identified in *mre11A470T ctf18▵* mutants. The first, Type I, rescues telomere attrition by invasion into longer telomeres using Rad51-dependent break-induced replication (BIR), producing two long telomeres. The second, Type II, consists of discrete fragments and appears to be mechanistically distinct from BIR given the lack Rad51-dependence of Type II survivors [51].

**Figure 10:**
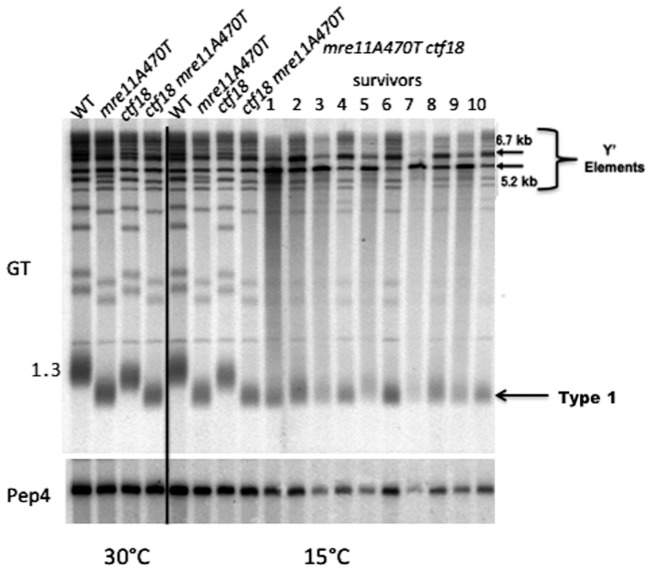
*mre11A470T ctf18*▵ cold sensitive survivors arise via recombinational mechanisms. [**GT**] DNA isolated from the indicated strains (top) grown at 30°C and 15°C and from ten independent survivors (1–10) were digested with XhoI and Southern analysis performed using poly GT as a telomeric probe. Arrow on right indicates position of Type I survivor. Note that the smearing of the distribution that represents the distribution of Type II-like survivors. [**PEP4**] Southern blot from above was stripped and probed with *PEP4* sequences as an internal loading control.

After the brief senescence of *mre11A470T ctf18▵* double mutants at 15°C, *RAD52*-dependent survivors arise that are most similar to Type I events, with the bulk of telomeres slightly larger than the presumed limit size allowing growth of cells (**Figure 10** **)**. Larger telomeres were also present as a highly diffuse but faint heterogeneous species, suggesting some form of Type II-like recombination. Extended growth in liquid media gives rise to the same diffuse pattern. Our findings suggest that the known Type I recombinational pathway is the major mechanism for survival in *mre11A470T ctf18▵* cells. Attempts to generate survivors in the absence of Rad52 were unsuccessful.

### Recombinational survivors of *mre11A470T ctf18▵* cold sensitivity are phenotypically reversible

We would predict that shifting cells to 30°C would restore Type I short telomeres to wild type, but that Type II-like telomeres would return to wild type only at a slower rate. If true, this prediction rules out any permanent changes to the telomeres at 15°C that would restrict the reactivation of telomerase.

**Figure 11:**
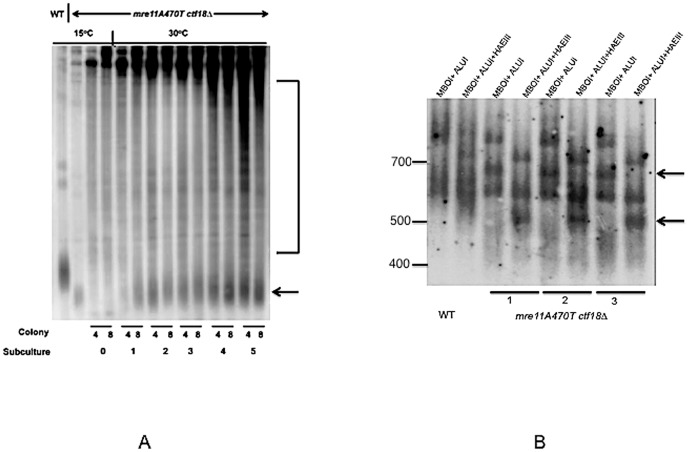
Survivors are Capable of Recovery at 30°C. [A] Two independent survivors of growth at 15°C (4,8) were shifted to growth at 30°C for multiple rounds of liquid subculturing. The DNAs were isolated and probed with polyGT to visualize the telomere size after Southern analysis. Control strains are shown in the left two lanes. [B] Three independent survivors (1–3) and wild type cells (WT) containing the HaeIII-templated telomerase were shifted from growth in YPD from 15°C to 30°C and subsequently grown at 30°C through five rounds of liquid subculturing. DNA was isolated from the terminal subculture at 30°C and digested with the 4-bp restriction enzymes, Alu1 and Mbo1, or with Alu1, Mbo1, and HaeIII. Top arrow on right points to the fragment in the left lane of strain 3 after double digestion without HaeIII that is not present after digestion with HaeIII and the bottom arrow refers to the a digestion product in the right lane of lane 3 not present in the left lane after digestion in the presence of HaeIII. Neither fragment is present in wild type cells.

Several criteria indicate that the original phenotype of *mre11A470T ctf18▵* can be recovered when the temperature of survivors are shifted from 15°C to 30°C. First, double mutants regain the ability to grow at 30°C. Second, telomeres return close to their pre-survivor size (**Figure 11A**). This finding is most consistent with recombinogenic ends that are also compatible with telomerase or other recombinational activities [35]. Third, the DDR pattern of Rad53 phosphorylation was qualitatively similar to the original strain grown at 30°C (**Figure 7**). Fourth, when cells that were shifted to 30°C were grown at 15°C, a mixture of cells are observed upon microscopic analysis. The general classes of cells include no growth, both irregular and circularly shaped micro-colonies, and full growth (**data not shown**). This finding is not easily consistent with the presence of a suppressor mutation present in each cell. Such cells would be expected to give rise to a uniform phenotype at 15°C and 30°C.

To detect the activity of telomerase *in vivo*, we used an allele of the RNA component of telomerase, TLC1 (*TLC1-1*), that contains a mutation within the RNA template region that introduces HaeIII sites into growing telomeres if telomerase is active [41]. The *TLC1-1* allele was integrated into wild type and *mre11A470T ctf18▵* cells, creating a tandem duplication of wild type and mutant *TLC1*. Our prediction was that the telomere elongation occurring on a shift of growth from 15°C to 30°C should allow HaeIII within the centromere-proximal regions of the telomere tract. In contrast, wild type cells are only be able to incorporate sites at the most terminal regions due to the slow turnover of telomeric terminal sequences under homeostatic conditions. To eliminate HaeIII fragments of less than 1 kb that represent internal GT tracts, we first digested the DNA with AluI and MboI. This digestion was carried out to reduce the background of unrelated fragments that may obscure the presence of telomeric HaeIII cleavage sites. We find HaeIII sites within terminal AluI or MboI fragments, after shifting from 15°C to 30°C for five rounds of liquid subculturing, but are not present in wild type strains grown under the same set of conditions (**Figure 11B**).

Analysis of the consensus sequences of the long and short Y’ subtelomeric elements that contain telomeric sequences between repeats (Ed Louis, personal communication) does not predict an AluI/MboI fragment that contains an internal HaeIII sites at consensus junctions, Given, the polymorphism of Y’ elements, however, it is not possible to rule out a contribution from Y’ elements.

## Discussion

We have presented several lines of evidence for the involvement of Ctf18 RFC in telomere replication, replication fork repair, and the initiation of cohesion at the telomere. This has been accomplished by a study of the genetic associations between Ctf18 and either telomerase or an Mre11 region located near the Mre11/Rad50 interface. From these data, we have constructed a more unified picture of Ctf18 – telomere interactions.

### The Role of Ctf18 in Telomerase Negative Cells

The question of the role of Ctf18 at the telomere is a part of a larger question: the inter-relationship between semi-conservative DNA synthesis, telomerase and recombination at the telomere. We therefore investigated the characteristics of Ctf18 in a telomerase-negative strain lacking the telomerase RNA (TLC1). Through an analysis of the phenotypes of *tlc1▵* and *tlc1▵ ctf18▵* mutant cells, we find that the absence of just one clamp loader, Ctf18 RFC, leads to a rapid (54 hour post germination) loss of growth and viability. In this regard, we cannot determine whether viability reflects cell death or cell cycle arrest. These data suggest that telomeres may require only a single clamp loader.

The *ctf18▵* mutation can result in the failure of Rad52, the essential homologous recombination protein, to repair double strand breaks [5]. Numerous studies have found that the absence of Rad52 also results in a fairly rapid loss of viability (54 hours post germination) in telomerase-negative strains. These data raised the possibility that *ctf18▵* and *rad52▵* fall into the same pathway under these conditions. However, the finding that *rad52▵ tlc1▵ ctf18▵* strains are synthetically lethal is inconsistent with this hypothesis. Our results therefore suggest at least two distinct but critical mechanisms that maintain telomere integrity: homologous recombination and repair of damage at the replication fork.

The critical function does not appear to be related directly to telomere size since *tlc1▵ rad52▵* and *tlc1▵ ctf18▵* have very similar sizes of about 100 bp after 36–54 hours of growth after germination. Although not observed, we cannot rule out the possibility of small amounts of single or double stranded DNA or of intermediates beyond the level of our detection by Southern analysis of uncut DNA. In contrast to Rad52-dependent events, defects in *CTF18*, encoding a clamp loader would be expected to have major defects in replication fork stalling and breaking as well as in mutagenesis during replication. A stalled replication fork can lead to reinitiation (restarting) of DNA replication even within a single cell cycle. Mre11 and Ctf18 may also facilitate telomeric DNA repair after fork breakage or mutagenesis through either the ATM or ATR checkpoint pathway.

Another difference between the Rad52 and Ctf18 pathways is the *tlc1▵ ctf18▵-*specific retention of a low level of viable cells throughout subculturing until the emergence of survivors. Indeed, this viable population is the likely source of survivor strains. Survivor strains in the double mutant emerge later than *tlc1▵* cells alone. It is possible that more generations are required in the double mutant to generate survivors. Alternatively, if a defined subset of viable cells remains phenotypically unique in *tlc1▵ ctf18▵*, viable single and double mutant cells could have a similar generation time.

Comparisons of *tlc1▵* and *tlc1▵ ctf18▵* survivors show a similar Type II-phenotype pattern of multiple large telomere fragments. However, there is more regularity in the size of the fragments produced in *tlc1▵ ctf18▵* survivors, compared to *tlc1▵* strains, possibly due to the additional replication-based activities in the presence of wild type Ctf18.

An alternative possibility is that the patterns of gene expression are substantially altered. The mere loss of telomerase activity, but not the telomere, activates a unique set of pathways as recently demonstrated [52] A further investigation of this altered pattern is clearly required. However, the induction or repression of specific gene products in our strains cannot easily explain the results presented in this study.

### The Interaction Between Ctf18 and Mre11

The multiple phenotypes of *mre11A470T*, in the absence of Ctf18, reflect the large range of genetic interactions, particularly in S phase. It is intriguing that many of the genetic interactions between *mre11A470T* and *ctf18▵* are synergistic. That is, the combinatorial presence of the *mre11A470T* allele and *ctf18▵* allele are greater than the additive effect of the phenotype conferred by each individual mutation. We found several instances of this phenomenon.

First, the two mutants, *mre11A470T* and *ctf18▵* both shorten telomere length, but clearly do not fall into a single epistasis group displaying one of the two phenotypes. As a consequence, the phenotype of *mre11A470T ctf18▵* is more extreme and is just slightly larger than the length of dysfunctional telomeres. At 15°C, this state is unstable leading to a rapid cold sensitive loss in both growth rate and viability. The major difference at the two temperatures is that the viability and telomere sizes of the cultures are stable at 30°C, but unstable at 15°C. These data suggest that Mre11A470T and Ctf18 proteins act in different pathways on the same substrate to provoke cold sensitive growth and loss in viability**.**

Second, the *mre11A470T ctf18▵* mutation confers a synergistic hypersensitivity to HU, a standard agent of stress at the replication fork due to the presence of a low dNTP pool size. HU treatment leads to fork cleavage and/or stalling [48]. Similarly, agents acting during replication such as MMS also confer an enhanced sensitivity to mutagenesis in the double mutant, compared to single mutants.

Third, the defective *mre11A470T ctf18▵* telomere separation during anaphase [9] has far lower levels of sister chromatid telomere separation than in *mre11A470T* or *ctf18▵* single mutants alone. This synergism suggests at least two means to regulate a common pathway for cohesion initiation.

Fourth, the *mre11A470T ctf18▵* double mutant phenotypes suggest that Mre11A470T and Ctf18 are in separate pathways acting on the telomere. For example, the *mre11A470T* allele could be aberrant for recruiting DSBs into the Tel1/ATM pathway. If Mre11A470T/Rad50 stability and/or conformation are altered, then the ability of Tel1 to bind to the telomere might be compromised and the homeostatic sizing control may be disrupted. Alternatively, the downstream activity of Mre11 in the Tel1 pathway may be disabled for the association with and facilitation of nuclease/helicase activities such as Sae2, Sgs1, Dna2, and Exo1 [24]–[27]. In either case, the function of Mre11 may lead to short telomeres that would not be able to use telomerase to establish a homeostasis in the absence of another function, Ctf18 that may be compromised for telomere size possibly due to defects at the replication fork. Consequently, the *mre11A470T ctf18▵* cells might senesce quickly with critical shortening of telomeres at the restrictive temperature.

Fifth, the type of DDR elicited by the ctf18▵ and mre11A470T mutants only share the common Rad53 phosphorylation step in ATM and ATR pathways [10]. Both mutants, however, have unique properties. While the *ctf18▵* mutant confers constitutive activation at 15°C and 30°C, phosphorylation of Mre11A470T only takes place at 15°C. In contrast, there is little phosphorylation in the double mutant at 30°C or 15°C. This suggests that Mre11A470T and Ctf18 elicit different classes of damage response. The loss of other repair functions may also lead to the Rad53 activation. This does not include the null allele of Mre11, which is required for upstream and downstream activities of the Tel1 pathway [53].

Sixth, previous data have led to the hypothesis that the S-phase role of cohesion acts by initiating, but not maintaining cohesion through interacting telomeres [9]. A defect in Ctf18 RFC should give rise to a failure of S phase-initiation of sister chromatid cohesion as well as a lack of sister chromatid separation [9]. The misrouting of damaged telomere DNA or the failure of Mre11 to act in the helicase/nuclease functions in Mre11 may contribute to improperly attached telomeres and a lack of cohesion in double mutants, leading to the synergistic behavior. Our data is consistent with the hypothesis that Ctf18 is normally involved in the initiation, but not maintenance, of telomere associations [9].

Finally, these data and the behavior of Ctf18 in telomerase-negative cells imply that Ctf18 is the primary PCNA clamp loader acting in semi-conservative replication at the telomere. Supporting this damaged replication fork-based model, a similar cold-sensitive pattern is observed in *mre11A470T pol32* mutations. Both Mre11 and telomerase are required for full Ctf18 function. It is compelling to speculate that both pathways have a similar function, possibly in the absence or limitation of telomerase.

We propose, therefore, that most of the phenotypes that we observe can be explained as the effects of defects in proteins involved in the repair of two types of damage: Mre11A470T, acting through misprocessing of broken and damaged DNA, and Ctf18, needed for the stability of the replication fork at the telomere. Possibly, Ctf18 RFC may be the sole clamp loader of PCNA in telomere sequences.

Curiously, among survivors of *mre11470T ctf18*▵ double mutants we observed Type I losses in telomere tract size that are compensated by BIR-mediated recombination bringing the telomeric tract to sizes sufficient for maintaining viability. What is mechanistically less clear are the Type II-like large heterogeneous telomere tracts that are also observed. There is some precedent for this diffuse pattern in *tlc1▵ cdc13▵* double mutants [54]. In addition, this type of diffuse pattern is typical of telomerase RNA mutants in *Kluyveromyces lactis*, a process that has been proposed to be the consequence of rolling circle replication [55].

All survivors in our study are recombinational in nature given their dependence on Rad52. However, this study does not demonstrate that telomerase is absent in survivors. Telomerase might simply not be sufficiently active to maintain functional telomeres. It is even conceivable that under some conditions different cells in a population can use Type I and Type II-like pathways as well as telomerase. Given the ability of survivors to use telomerase one possible interpretation is a shift from a recombinational to telomerase mechanisms in the shift of survivors from growth at 15°C to 30°C, with telomerase possibly being maintained in active and inactive states. Alternatively, survivor strains may contain both recombinational and telomerase means of telomere elongation in the same cell or population of cells. It is possible that we may need to consider multiple mechanisms in a given cell or at a particular telomere as serious alternatives in future experiments.

In summary, we have presented data to support the hypothesis that Ctf18RFC and Mre11 act at the semi-conservative portion of telomere replication where they both play required roles in telomere integrity through the effects of two types of damage, one (Ctf18) in permitting telomere-specific replication fork movement by interaction with PCNA, and the second (Mre11) directly involved in ATM and ATR processing of replication fork-initiated DNA damage, telomerase activity, or telomere capping. This defect, we believe, is the source of the many phenotypes observed in this absence of both proteins. The effects that we observe at the telomere in *mre11A470T ctf18▵* mutations are likely to affect other genomic sites given the sensitivity of strains to HU and MMS. However, the mechanisms conferred by these mutations and their similarities to the telomere are issues that will be answered in the future.

## Supporting Information

Figure S1 Viability of *tlc1▵ ctf18▵* Cells. Ten microliters of wild type, *ctf18▵*, *tlc1▵* and *tlc1▵ ctf18▵* cell cultures after dilution at each stage of subculturing to from 10^−1^ to 10^−4^ fold. The number of viable cells were counted from an appropriate dilution and adjusted to the number of viable cells/ml. In this graph, the number of viable cells was normalized to the wild type value and plotted as the relative fold decreases in viability in viability as a function of the time of subculturing as describe in Materials and Methods.
(TIF)

Figure S2 *tlc1▵* and *tlc1▵ ctf18▵* survivors exhibit Type I- and Type II-like amplification. Subcultured *tlc1▵* and *tlc1▵ ctf18▵* survivors after 196 hours of subculturing were sub-cloned on YPD and each subclone was grown for 18 hours at 30°C. Nine independent *tlc1▵* and ten *tlc1▵ ctf18▵*colonies were inoculated into 5 ml YPD and grown for 18 hours at 30°C. Genomic DNA was isolated and digested with XhoI followed by Southern analysis using poly [GT] as a probe.
(TIF)
