# Supplementary figures and images for: The Ctf18RFC Clamp Loader Is Essential for Telomere Stability in Telomerase-Negative and mre11 Mutant Alleles
Source: PLoS One. 2014 Feb 12;9(2):e88633. doi: 10.1371/journal.pone.0088633 (PMC3923045; doi:10.1371/journal.pone.0088633)

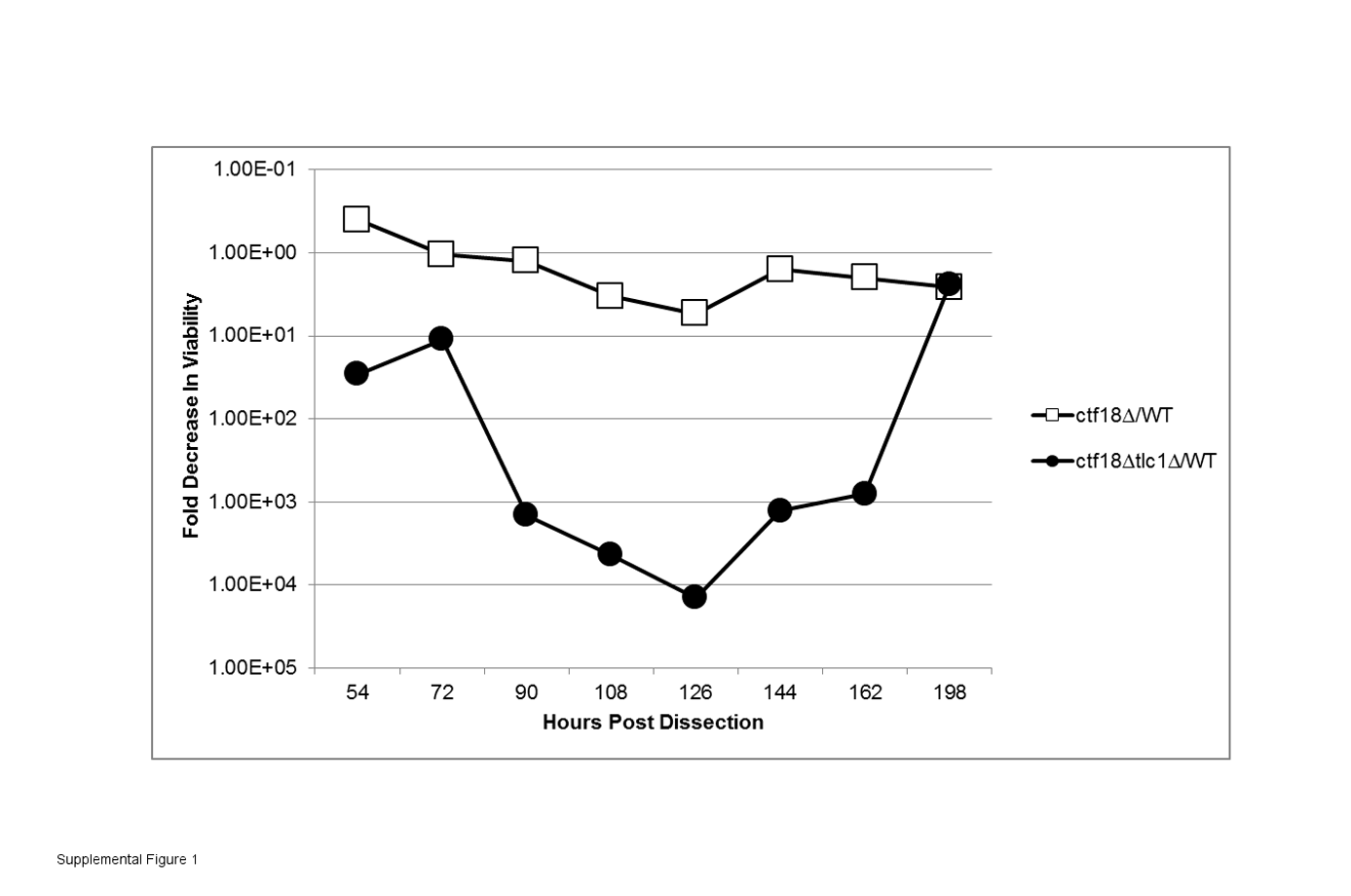

Supplement: Figure S1 — Viability of tlc1▵ ctf18▵ Cells. Ten microliters of wild type, ctf18▵, tlc1▵ and tlc1▵ ctf18▵ cell cultures after dilution at each stage of subculturing to from 10−1 to 10−4 fold. The number of viable cells were counted from an appropriate dilution and adjusted to the number of viable cells/ml. In this graph, the number of viable cells was normalized to the wild type value and plotted as the relative fold decreases in viability in viability as a function of the time of subculturing as describe in Materials and Methods. (TIF) [file pone.0088633.s001.tif]

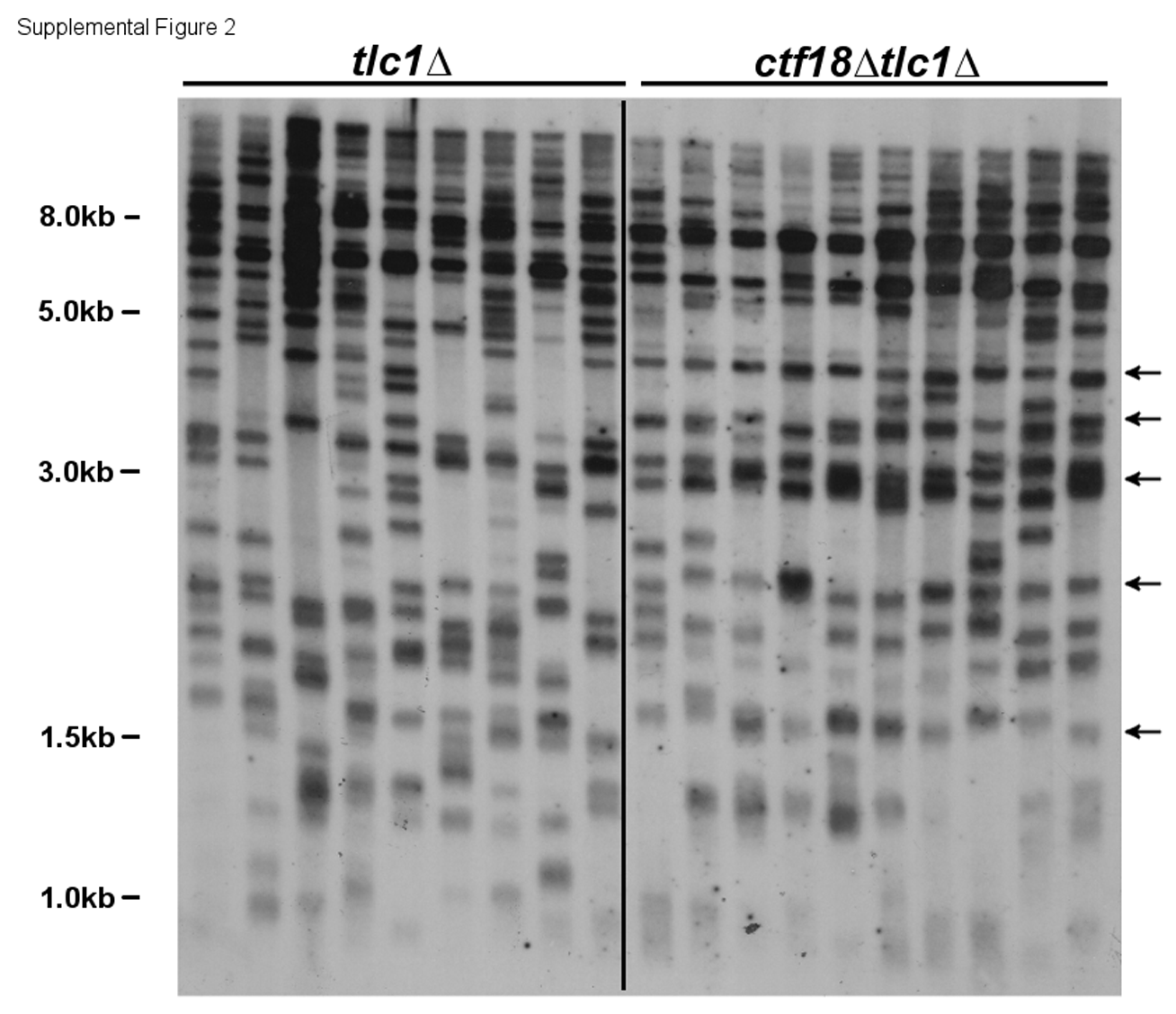

Supplement: Figure S2 — tlc1▵ and tlc1▵ ctf18▵ survivors exhibit Type I- and Type II-like amplification. Subcultured tlc1▵ and tlc1▵ ctf18▵ survivors after 196 hours of subculturing were sub-cloned on YPD and each subclone was grown for 18 hours at 30°C. Nine independent tlc1▵ and ten tlc1▵ ctf18▵colonies were inoculated into 5 ml YPD and grown for 18 hours at 30°C. Genomic DNA was isolated and digested with XhoI followed by Southern analysis using poly [GT] as a probe. (TIF) [file pone.0088633.s002.tif]
